# Dance of the Sugar: Two Case Reports of Chorea Associated With Nonketotic Hyperglycemia

**DOI:** 10.7759/cureus.36254

**Published:** 2023-03-16

**Authors:** Rafaela C Pereira, Fábio Caleça Emidio, Angelo Dias, Rosário Blanco Saez, Motasem Shamasna, Joana Pestana

**Affiliations:** 1 Department of Internal Medicine, Centro Hospitalar Universitário do Algarve - Hospital de Faro, Faro, PRT; 2 Department of Radiology, Centro Hospitalar Universitário Lisboa Norte, EPE, Lisboa, PRT; 3 Department of Neurology, Centro Hospitalar Universitário do Algarve - Hospital de Faro, Faro, PRT

**Keywords:** neuroimaging, basal ganglia, hemichorea/hemiballism, diabetes mellitus, non-ketotic hyperglycemia

## Abstract

Hyperglycemia-induced chorea/ballism is a rare clinical entity that often occurs in the setting of nonketotic hyperglycemia due to poor glycemic control in elderly patients with a diagnosis of type 2 diabetes mellitus (DM). This condition is typically characterized by hemichorea/hemiballism and unique brain imaging findings in the contralateral basal ganglia. Treatment involves the correction of blood glucose, and most cases resolve without additional therapy. Here we report two cases of this condition in which patients with type 2 DM presented with nonketotic hyperglycemia and typical neuroimaging findings. Although rare, clinicians should be aware of this condition in patients with diabetes who present with sudden abnormal movements since its prompt diagnosis and treatment often lead to a favorable outcome.

## Introduction

Chorea/Ballism is a rare complication of hyperglycemia in patients with diabetes mellitus (DM) [1]. Most patients present with nonketotic hyperglycemia associated with hemichorea/hemiballism and hyperintensity within the contralateral basal ganglia in T1-weighted MRI images [2], hence known as hyperglycemia-induced hemichorea-hemiballism, with only a small percentage of cases showing bilateral manifestations and ketosis [3,4]. The exact pathophysiological mechanism underlying this condition is still unclear. The current hypothesis proposes that chorea/ballism is attributable to hyperglycemia-induced basal ganglia dysfunction, likely due to the stimulation of the anaerobic pathways in the brain and, consequently, inhibition of the Krebs cycle [5]. The primary treatment involves correcting hyperglycemia. Most cases successfully improve over days to weeks without the need for further treatment, with only a few cases demanding additional symptomatic therapy with anti-chorea medication [2,5]. Herein, we describe two cases of chorea/ballism induced by nonketotic hyperglycemia in patients with type 2 DM. Our purpose in presenting these two cases is to draw clinicians' attention to this condition because, despite its rarity, the high prevalence of diabetes worldwide increases the likelihood of encountering this condition. While the entity discussed in these cases is not novel and has been described in the literature, it is still not well-known.

## Case presentation

Case 1

**Figure 1:**
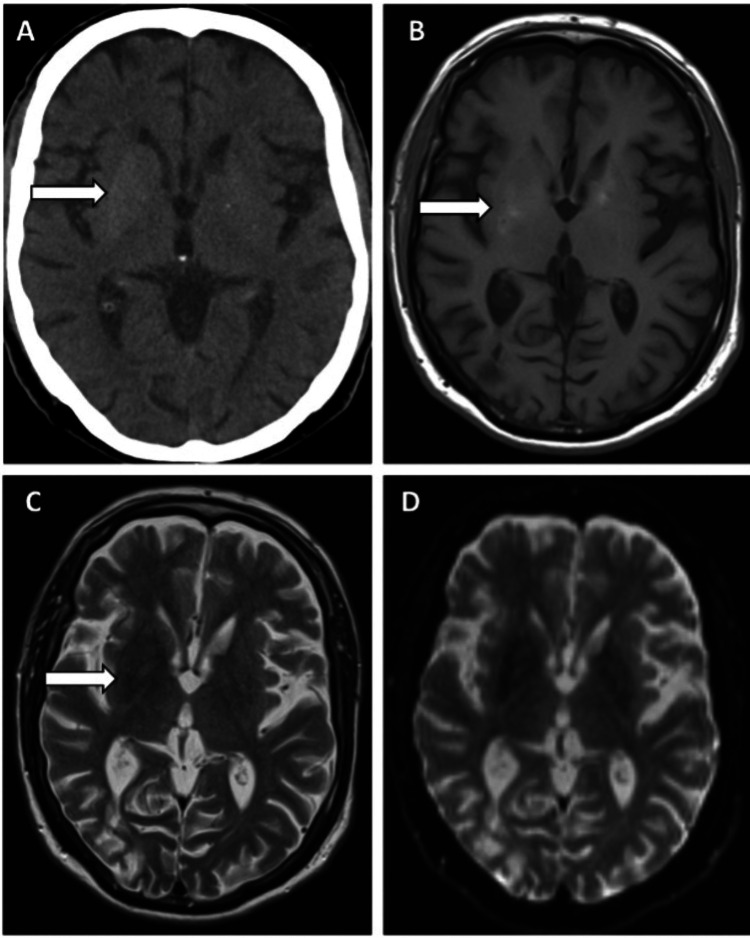
Brain images of case 1. A. Axial noncontrasted brain CT scan on admission demonstrates faint and homogeneous increased density of the right lentiform nucleus (LN; arrow). This density alteration respects the expected anatomy being confined to the LN without marked distortion. No signs of mass effect or edema. In the remaining parenchyma, there were no signs of acute intracranial lesions. B. Axial noncontrasted T1-weighted spin-echo MRI image of the brain demonstrates a slight increase of signal intensity within the right LN, predominantly along the posterior aspect, partially corresponding to the area of hyperdensity seen on the preceding CT exam. C. Axial T2-weighted turbo spin echo MRI shows a slight decrease of signal within the right putamen (white arrow). D. Axial diffusion-weighted image shows no areas of restriction of the diffusion of water molecules within the basal ganglia to suggest recent ischemia within the corresponding areas of T1 and T2 signal change.

A 72-year-old man recently diagnosed with type 2 DM medicated with metformin and vildagliptin presented to the hospital with a four-day history of involuntary left upper- and lower-limb movements. He had no history of other relevant medical conditions. On the neurological examination, the patient was fully alert and oriented, with clear speech, but had a rapid swing of his left upper- and lower extremities. No further deficits in the neurological examination were found. Laboratory tests revealed that his random blood glucose level was 296 mg/dL, pH 7.35, bicarbonate 22.6 mEq/L, and anion gap was 12.6. The fasting blood glucose level was 369 mg/dL with a glycated hemoglobin A1c (HbA1c) of 16.1%. Noncontrasted cranial CT obtained at admission showed mildly increased density within the right lentiform nucleus without evidence of acute intracranial pathology in the remaining brain parenchyma (Figure 1A). Subsequently, a brain MRI scan was performed, demonstrating a corresponding slight hyperintense signal in the right lentiform nucleus on T1-weighted images (Figure 1B) and slight hypointense on T2-weighted images signal intensity (Figure 1C). Diffusion-weighted images revealed normal signal intensity in the left putamen (Figure 1D). Gradual improvement of the patient's symptoms was observed due to blood glucose control with insulin glargine. After eight days of treatment, the patient was discharged with a prescription for 30 international units of glargine, as well as metformin and vildagliptin.

Case 2

**Figure 2:**
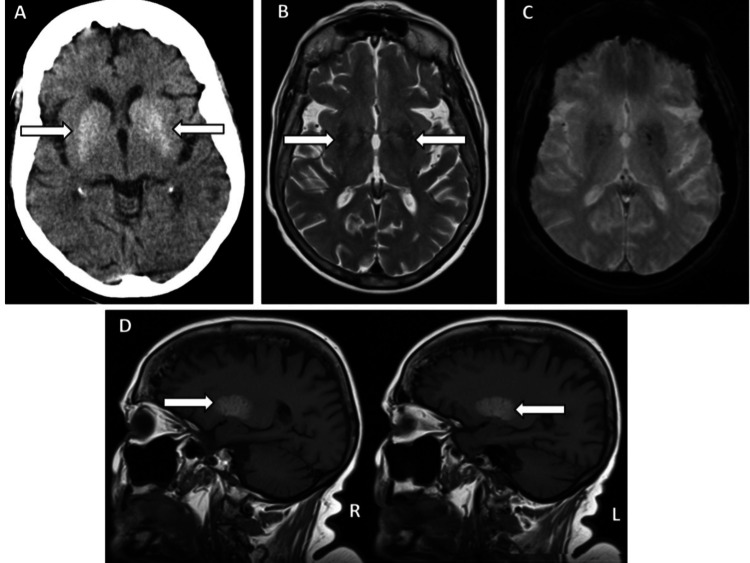
Brain images of case 2. A. Axial noncontrasted brain CT scan on admission demonstrates bilateral, symmetric, homogeneous hyperattenuation of lentiform and caudate nuclei. This density alteration respects the expected anatomy being confined to the basal ganglia without marked distortion. No signs of mass effect or edema. In the remaining parenchyma, there were no signs of acute intracranial lesions. B. Axial T2-weighted turbo spin echo MRI demonstrates a decrease of signal in both putamens (arrows). C. Axial diffusion-weighted image shows no areas of restriction of the diffusion of water molecules within the basal ganglia to suggest recent ischemia within the corresponding areas of T1 and T2 signal change. D. Left (L) and right (R) sagittal noncontrasted T1-weighted spin-echo MRI images of the brain show bilateral and symmetric increased signal intensity in the putamen (arrows).

A 76-year-old woman with a history of hypertension, obesity, and type 2 DM, poorly controlled despite metformin treatment and 16 international units of insulin glargine, presented to the hospital. She was brought to the hospital with a two-week history of continuous, involuntary, and non-rhythmic movements involving the four limbs. On the neurological examination, the patient demonstrated periodic choreiform and ballistic movements without any cranial nerve deficit. Her pupils were isochoric, and her light reflex was bilaterally positive. Her speech was confused and dysarthric. Deep tendon reflexes were normal, and she had no motor or sensory deficit. Laboratory tests revealed her random blood glucose level of 263 mg/dL, pH 7.44, bicarbonate 25.9 mEq/L, and an anion gap of 9.0. Fasting blood glucose was 417 mg/dL, and HbA1c was 16.5%. Liver tests and electrolyte values were normal. Noncontrasted cranial CT at admission demonstrated increased density in the basal ganglia (caudate and lentiform nuclei) (Figure 2A). Brain MRI showed corresponding symmetric and bilateral hyperintense T1 signal (Figure 2D) and hypointense T2 signal in the putamen (Figure 2B). Diffusion-weighted images revealed normal signal intensity in the basal ganglia (Figure 2C). The patient's condition showed significant improvement after achieving glycemic control by increasing the dosage to 62 units of insulin glargine per day. After a 12-day hospitalization, the patient was discharged and resumed her normal activities.

None of these patients experienced a recurrence of symptoms. They are currently undergoing regular follow-ups to ensure that glucose levels are properly controlled.

## Discussion

The presented cases illustrate the occurrence of chorea/ballism induced by nonketotic hyperglycemia. The diagnosis was based on a combination of clinical features, laboratory tests, and imaging studies [4]. Both patients had no prior history of involuntary movements and presented at admission with nonketotic hyperglycemia. In the first case, the patient was recently diagnosed with type 2 DM and presented with rapid movement of his left limbs. In contrast, the patient of the second case presented with a history of poorly controlled diabetes and two weeks of continuous, involuntary, and non-rhythmic movements involving all four limbs. Both cases showed characteristic imaging findings typified by hyperdensity on CT scan and corresponding increased T1 signal and decreased T2 signal on MRI within the basal ganglia. One of the cases showed bilateral involvement of the basal ganglia, which is a rare manifestation of this condition, reported in only 10% of the cases [4]. Bilaterally increased T1 signal intensity within the basal ganglia should lead to consideration of other metabolic (e.g., hepatic encephalopathy, disorders of calcium metabolism), toxic (e.g., manganese, carbon dioxide, and methanol poisoning) or neurodegenerative disorders (Wilson disease) [6]. However, chorea/ballism not related to hyperglycemia has a different presentation than the patient in case 2. These alternative diagnoses can be largely excluded based on patient history and laboratory findings. The clinical course of chorea/ballism secondary to nonketotic hyperglycemia is usually favorable after the correction of hyperglycemia. Treatment involves tightly controlling glucose blood levels, usually with insulin therapy. The goal is rapidly correcting the hyperosmolar state, which tends to resolve symptoms within hours to weeks. Both cases described in this report showed relief from choreic symptoms following treatment with insulin glargine and glycemic control.

## Conclusions

In summary, nonketotic hyperglycemia chorea/ballism is a rare but essential condition that clinicians should be aware of, especially given the high prevalence of DM. Patients presenting with new-onset acute or subacute chorea/ballism, particularly if unilateral, should be evaluated for hyperglycemia regardless of a known diagnosis of diabetes. Prompt recognition and management of hyperglycemia-induced chorea/ballism are crucial, as this condition can be easily treated by correcting blood glucose levels, leading to the complete resolution of symptoms. Furthermore, since nonketotic hyperglycemia chorea/ballism can be the first manifestation of undiagnosed diabetes mellitus, clinicians should consider this condition a potential diagnostic clue. Overall, these cases underscore the importance of recognizing and managing nonketotic hyperglycemia chorea/ballism, a rare but easily treatable condition that can have a significant impact on a patient's quality of life.
